# Relations Between Oxidized Low-density Lipoproteins and Fat-soluble Vitamin Concentrations in Obese Children – Preliminary Study

**DOI:** 10.34763/devperiodmed.20172103.266271

**Published:** 2017-10-28

**Authors:** Joanna Gajewska, Jadwiga Ambroszkiewicz, Halina Weker, Magdalena Chełchowska

**Affiliations:** 1 Screening Department Institute of Mother and Child in Warsaw, Warsaw Poland; 2 Department of Nutrition Institute of Mother and Child in Warsaw, Warsaw Poland

**Keywords:** fat-soluble vitamins, oxidized LDL, prepubertal period, witaminy rozpuszczalne w tłuszczach, oksydowane LDL, okres przedpokwitaniowy

## Abstract

**Introduction:**

Although lipid peroxidation products are formed during normal cell metabolism, they appear mostly in pathological conditions via producing an excess of free radicals that can react with unsaturated fatty acids, in particular low-density lipoprotein (LDL).

The aim of this study was to investigate the relations between oxidized LDL (oxLDL), the fat-soluble vitamin status and the anthropometric parameters in prepubertal obese children.

**Material and methods:**

Thirty-two obese (SDS-BMI >2) and 25 non-obese children (SDS-BMI <-1+1>) were included in the study. The concentration of oxLDL was determined in the serum by the ELISA assay. Vitamin A and E were measured by the high-pressure liquid chromatography method. Total cholesterol, LDL- and HDL-cholesterol, and triglyceride levels were determined by enzymatic methods.

**Results:**

The concentrations of oxLDL and vitamin A were higher in obese children than in normal-weight controls by about 50% (p=0.01) and 40% (p=0.001), respectively. In obese children the significant positive correlation was found between oxLDL and vitamin A concentrations (p<0.05). In addition, oxLDL correlated positively with BMI values (p<0.05) and the amount of fat mass (kg) (p<0.02) in these patients. Concentrations of vitamins A and E correlated with the level of total cholesterol (p<0.05; p<0.01, respectively). Moreover, a positive correlation between vitamin E and LDL-cholesterol was observed (p<0.05).

**Conclusions:**

Our preliminary study shows that oxLDL starts early during the prepubertal period and may precede atherosclerotic lesions. We suggest there is an occurrence of relationships between vitamin A and oxidized LDL in prepubertal obese children. Vitamin A and E concentrations are also associated with dyslipidemia in these patients.

## Streszczenie

**Wprowadzenie:**

Produkty peroksydacji lipidów powstają w czasie fizjologicznych przemian zachodzących w komórkach. Jednakże, znacznie częściej pojawiają się w stanach patologicznych, gdy nadmiar wolnych rodników reaguje z nienasyconymi kwasami tłuszczowymi szczególnie lipoprotein o niskiej gęstości (LDL).

Celem pracy było zbadanie zależności pomiędzy oksydowanymi LDL (oxLDL), statusem witamin rozpuszczalnych w tłuszczach a parametrami antropometrycznymi u dzieci otyłych w okresie przedpokwitaniowym.

**Materiał i metody:**

Grupę badaną stanowiło 32 dzieci otyłych (SDS-BMI >2) oraz 20 dzieci z prawidłową masą ciała (SDS-BMI <-1+1>). Stężenie oxLDL oznaczano w surowicy za pomocą testu ELISA. Witaminę A i E mierzono metodą wysokociśnieniowej chromatografii cieczowej. Poziom całkowitego cholesterolu, cholesterolu LDL i HDL oraz poziom triglicerydów oznaczano metodami enzymatycznymi.

**Wyniki:**

Stężenie oxLDL i witaminy A było wyższe u dzieci otyłych niż w grupie kontrolnej odpowiednio o około 50% (p=0,01) i 40% (p=0,001). U dzieci otyłych stwierdzono istotną dodatnią korelację między stężeniem oxLDL a stężeniem witaminy A (p<0,05). Ponadto, stężenie oxLDL korelowało pozytywnie z wartościami BMI (p<0,05) i ilością tkanki tłuszczowej (kg) (p<0,02) u tych pacjentów. Stężenia witamin A i E korelowały z poziomem cholesterolu całkowitego (odpowiednio p<0,05, p<0,01). Oprócz tego zaobserwowano dodatnią korelację pomiędzy witaminą E i cholesterolem frakcji LDL (p<0,05).

**Wnioski:**

Wstępne badanie wykazały, że wzrost stężenia oxLDL pojawia się u dzieci otyłych już w okresie przedpokwitaniowym i może poprzedzać u nich zmiany miażdżycowe. Uzyskane wyniki sugerują występowanie zależności pomiędzy witaminą A i oxLDL u tych pacjentów. Stwierdzono też związki pomiędzy stężeniem witaminy A i E we krwi a dyslipidemią u dzieci otyłych w okresie przedpokwitaniowym.

## Introduction

According to many studies, oxidant-antioxidant balance is needed to prevent freeradical-induced damage in nucleic acids, proteins and lipid structures. An important consequence of disturbances in the oxidant-antioxidant balance is lipid peroxidation [1]. Although lipid peroxidation products are formed during normal cell metabolism, these appear mostly in pathological conditions via producing an excess of free radicals that can react with unsaturated fatty acids, in particular low-density lipoprotein (LDL) [2]. In adults, higher oxLDL concentrations have been associated with higher body mass index (BMI), body fat, and insulin resistance [3, 4, 5]. Oxidation of this protein is considered a key factor in the pathogenesis of the metabolic syndrome, atherosclerosis and cardiovascular disease (). Elevated oxidized LDL (oxLDL) concentrations were observed mostly in obese adults, but much less is known about its relation to obesity in childhood [6, 7, 8]. In addition, obesity in adults and children is associated with an inflammatory condition that correlates with oxidative stress and may cause greater utilization of antioxidants [9].

Fat-soluble vitamins, which act as free radical scavengers, belong to the most important components of the antioxidant defense of lipids. It is suggested that the process of reducing oxidative damages may require higher concentrations of antioxidants, such as vitamin A and vitamin E [10]. Vitamin A (retinol) is a micronutrient required for growth and development, conceivably affecting lipid metabolism, energy regulation and body composition [11]. Vitamin E is most commonly found in the form of alpha-tocopherol and acts as a peroxyl scavenger and thus prevents the oxidation of membrane lipids [12]. Given the importance of these vitamins in lipid metabolism, there is also a need to investigate the relationship between fat-soluble vitamins and lipid peroxidation products in obesity. Recent studies in young populations suggest that fat deposition and chronic inflammation are associated with deficiencies in concentrations of fat-soluble vitamins [13, 14, 15]. Other studies show positive relations between levels of these vitamins and measures of obesity [16]. Thus, research data concerning these associations in obese children are inconsistent.

Therefore, the aim of this study was to investigate the relations between oxidized low-density lipoproteins, fat-soluble vitamin status and anthropometric parameters in prepubertal obese children.

## Methods

Thirty-two obese children (age, 7.8±1.5 years; boys, 47%) were included in the study. Healthy normal-weight children (n=25) were the reference group. Obese and non-obese children who were taking medications that could affect growth, pubertal development, nutritional status or dietary intake were not included. Physical examination was performed and body mass index (BMI) was calculated. Children were classified as obese (SDS-BMI >2) and non-obese (SDS-BMI <-1+1>). Body composition was measured by dual-energy X-ray absorptiometry (DXA) using Lunar Prodigy (General Electric Healthcare, UK) with pediatric software 9.30.044.

We assessed the average daily energy intake and the percentage of energy intake from protein, fat and carbohydrates in the diets of obese and non-obese children. Patients had 3-5 meals every day. The dietary intake and physical activity data were collected using randomly selected 3-day records. Average daily food rations and their nutritional value were calculated using nutritional analysis software (Dieta5^®^) [17].

Venous blood samples were collected between 8.00 and 10.00 in the morning after overnight fasting and centrifuged (1000g for 10 min at 4^o^C). The concentration of oxLDL was determined in the serum by the ELISA assay (Immundiagnostik AG, Germany). Vitamins A and E in the serum were determined by the high-pressure liquid chromatography method (HPLC), (Knauer, Germany) [18]. Total cholesterol, LDL- and HDL-cholesterol and triglyceride levels were measured by enzymatic methods (ROCHE, Switzerland).

This study had been approved by the Ethics Committee of the Institute of Mother and Child. Informed consent was obtained from the parents of all the examined children.

Statistical analysis was performed using Statistica version 8.0. The results are presented as mean±standard deviation (SD) for normally distributed data or medians and interquartile range (25^th^-75^th^ percentiles) for non-normally distributed variables. The Kolmogorov-Smirnov test and graphical inspections of data were used for evaluating the distribution for normality. Differences in anthropometric characteristics and biochemical parameters of obese and non-obese children were assessed using the Student’s t-test for normally distributed data and the non-parametric Mann-Whitney test for non-normally distributed variables. Spearman or Pearson correlations between parameters were calculated. Data are presented with p<0.05 considered statistically significant.

## Results

As expected, there are significant differences in the body weight, BMI and body composition between obese and non-obese children of the same age (tab. I). Average BMI was about 50% (p<0.001) higher in obese patients than in controls. The obese children had a fourfold greater (p<0.001) fat mass and 2-fold higher percentage of fat mass (p<0.001) compared with non-obese subjects. Moreover, obese patients had greater lean tissue mass and total BMC by approximately 30% (p<0.001). The daily energy intake of obese children before intervention was higher (p<0.05) compared with the controls. However, the proportions of protein, fat and carbohydrates in the daily energy intake were similar in both groups (tab. I).

The concentrations of oxLDL and vitamin A were higher in obese children than in normal-weight controls by about 50% (p=0.01) and 40% (p=0.001), respectively (tab. II). No difference was observed in the concentration of vitamin E between both groups. The level of HDL-cholesterol was lower (p=0.001), while the level of triglyceride was higher (p=0.01) in obese children than in controls.

A significant positive correlation was found between oxLDL and vitamin A concentrations (p<0.05) in obese children (tab. III). In addition, oxLDL correlated positively with BMI values (p<0.05) and the amount of fat mass (kg) (p<0.02) in these patients. No correlations were observed between oxLDL and lipid parameters.

Concentrations of vitamins A and E correlated with the level of total cholesterol (p<0.05; p<0.01, respectively). In addition, vitamin E correlated positively with LDL-cholesterol (p<0.05). No correlations were observed between fat-soluble vitamins and triglycerides, or HDL-cholesterol.

## Discussion

Oxidized low-density lipoprotein is one that may reflect the oxidative stress in organisms [19]. An increase in oxLDL concentrations was found in populations of obese children with a wide age range and different pubertal stages [7, 8, 20]. In this study, we showed higher concentrations of oxLDL associated with dyslipidemia including lower concentrations of HDL-cholesterol and higher concentrations of triglyceride in prepubertal obese children compared with normal-weight subjects. Similarly to our results, Okur et al. [8] observed higher values of oxidized protein related with disturbances in the lipid profile in obese children during the same pubertal stage. In addition, we observed positive relations between oxLDL and BMI, as well as the amount of fat mass (kg), but not the percentage of fat mass (%). It is suggested that greater adiposity, especially abdominal fat and low-extremity adiposity is associated with increased levels of oxidative stress. Furthermore, higher waist circumference, leptin and insulin resistance have been significantly linked with higher oxLDL levels in pediatric populations [8, 21]. These changes might be partially reversible by lifestyle intervention, even if patients do not reach normal body weight [22].

According to many authors, greater adiposity is associated with increased levels of oxidative stress, leading to reduced concentrations of antioxidants, including fat-soluble vitamins [9, 23]. These associations may result from differences in the energy intake and/ or sequestering of lipophilic vitamins in adipose tissue [24]. Lower values of fat-soluble vitamins in American and European populations of obese children compared with normal-weight controls were observed [25, 26]. In addition, it is suggested that the increased production of reactive oxygen species in obesity may lead to increased utilization and thus low availability of antioxidant enzymes and vitamins [27].

In opposition to these studies, we found higher concentrations of vitamin A in obese than in normal-weight children, but these values are still within the reference range (0.8-2.8 μmol/l) [28]. A significant association between serum retinol concentrations and adiposity was found by Gunanti et al. [15] in Mexican-American children 8-15 years of age. Garcia et al. [16] also observed positively associated vitamin A with BMI and abdominal fat mass in Mexican school-age children. In addition, serum retinol concentrations were elevated in overweight and obese Brazilian adolescents, while no differences in vitamin E concentrations were found between these groups [29]. It seems that the homeostatic regulation of vitamin A through storage in the liver and controlled release from the liver may provide tissue with optimal amounts of retinol, in this way protecting against the toxicity of this vitamin [15]. However, it is not impossible that the process of reduction of oxidative damages requires increased antioxidant concentrations, among them fat-soluble vitamins.

**Table I:** Anthropometric characteristics and dietary intake of obese and non-obese children. Tabela I. Charakterystyka wybranych cech antropometrycznych i udział podstawowych składników pokarmowych w średniej racji pokarmowej u dzieci otyłych i w grupie kontrolnej.

	Obese children *Dzieci otyłe*	Non-obese children *Dzieci z prawidłową masa ciała*	P-value *P*
Age (y)^a^ *Wiek (lata)*	7.8 ± 1.2	7.5 ± 1.5	0.479
Girls/Boys *Dziewczynki/Chłopcy*	17/15	13/12	
Weight (kg)^b^ *Masa ciała (kg) ^b^*	40.2 (36.7-49.7)	23.4 (19.8-26.7)	<0.001
BMI (kg/m^2^)^b^ *BMI (kg/m^2^)^b^*	23.4 (21.1-24.7)	15.3 (14.5 -15.9)	<0.001
**Body composition**: ***Skład ciała***:			
Fat mass (%)^a^ *Masa tłuszczowa (%)^a^*	40.7 ± 4.5	20.2 ± 6.3	<0.001
Fat mass (%)^b^ *Masa tłuszczowa (%)^b^*	15.8 (12.8-19.6)	4.0 (2.9-5.8)	<0.001
Lean mass (kg)^a^ *Masa mięśniowa (kg)*^a^	24.5 ± 3.0	18.4 ± 3.0	<0.001
Total BMC (kg)^b^ *Całkowite BMC (kg)*^b^	1.2 (1.0-1.4)	0.9 (0.7 -1.0)	<0.001
**Dietary intake: *Udział podstawowych składników pokarmowych***:			
Energy (kcal/d)^b^ *Energia (kcal/d)*^b^	1745 (1331-2075)	1540 (1240-1824)	0.032
Protein (% of energy intake)^b^ *Udział energii z białka (%)*	13.2 (12.1-15.0)	13.8 (11.5-14.6)	0.445
Carbohydrate (% of energy intake)^b^ *Udział energii z węglowodanów (%)*	53.5 (49.0-56.4)	52.2 (47.2-56.3)	0.548
Fat (% of energy intake)^b^ *Udział energii z tłuszczu (%)*	33.3 (30.6-36.8)	34.0 (29.4-39.5)	0.939

a Data are presented as mean values±SD; *Dane przedstawione jako wartości średnie±SD* ^b^Data are presented as median values (25^th^-75^th^ percentiles); *Dane przedstawione jako mediany (25-75 percentyl)* BMC, bone mineral content (*zawartość minerałów w kośćcu*)

**Table II:** Biochemical measurements in obese and non-obese children. Tabela II. Parametry biochemiczne u dzieci otyłych i w grupie kontrolnej.

	Obese children *Dzieci otyłe*	Non-obese children *Dzieci z prawidłową masą ciała*	P-value *P*
oxLDL(ng/ml)^b^	466 (313 – 658)	303(167-438)	0.010
Vitamin A (μmol/l)^a^	1.62 ± 0.46	1.15 ± 0.13	0.001
Vitamin E (μmol/l)^a^	18.5 ± 5.24	17.1 ± 1.76	0.238
LDL-cholesterol (mg/dl)^b^	104(94-117)	105(95-118)	0.799
HDL-cholesterol(mg/dl)^a^	49.7 ± 8.4	62.0 ± 9.3	0.001
Total-cholesterol(mg/dl)^a^	171 ± 20	177 ± 20	0.368
Triglycerides (mg/dl)^b^	79.0(59.8-129)	56.5(42.8-77.8)	0.010

a Data are presented as mean values±SD; *Dane przedstawione jako wartości średnie±SD* ^b^ Data are presented as median values (25^th^-75^th^ percentiles); *Dane przedstawione jako mediany (25-75 percentyl)*

**Table III:** Relationships between biochemical and anthropometric parameters in obese children. Tabela III. Zależności pomiędzy parametrami biochemicznymi i antropometrycznymi u dzieci otyłych.

	BMI *BMI*	Fat mass (%) *Masa tłuszczowa*	Fat mass (kg) *Masa tłuszczowa*	Total-cholesterol *Całkowity cholesterol*	LDL-cholesterol *LDL-cholesterol*	oxLDL *oxLDL*
oxLDL	0.360^*^	0.330	0.408^**^	-0.055	-0.008	-
Vitamin A	-0.003	0.040	0.030	0.381^*^	0.331	0.348^*^
Vitamin E	-0.280	-0.220	-0.257	0.448^***^	0.386^*^	0.128

* p<0.05; ^**^p<0.02; ^***^p<0.01

We observed positive correlations between oxLDL and vitamin A in prepubertal obese children. However, no relations between oxidized protein and vitamin E were obtained in these patients. Similar to us, Beck et al. [30] observed higher values of oxLDL in obese women, and did not find any association between oxidized LDL and vitamin E in obese subjects. Moreover, Albuquerque et al. [29] found that elevated serum retinol but not alpha-tocopherol concentrations were associated with dyslipidemia in overweight or obese adolescents. Relations between both vitamins and lipid parameters were showed by Garcia et al. [16]. These authors found positive relations in obese children between fat-soluble vitamins and triglycerides, as well as total cholesterol. In our previous and in presented studies we found disturbances in body composition and abnormal lipid profile in prepubertal obese children (31, 32). In this study we also found positive relations between vitamins A and E and total cholesterol and additionally between vitamin E and LDL-cholesterol in obese patients. We agree that higher concentrations of lipids may require higher concentrations of fat-soluble vitamins to prevent deficiencies of these vitamins and increased risk of cardiovascular diseases [16].

## Conclusion

Our preliminary study shows that oxLDL starts early during the prepubertal period and may precede atherosclerotic lesions. We suggest the occurrence of relationships between vitamin A and oxidized LDL in prepubertal obese children. Vitamin A and E concentrations are also associated with dyslipidemia in these patients.
